# Wear of the Charité^®^ lumbar intervertebral disc replacement investigated using an electro-mechanical spine simulator

**DOI:** 10.1177/0954411915576537

**Published:** 2015-03

**Authors:** Parshia Moghadas, Aziza Mahomed, Duncan ET Shepherd, David WL Hukins

**Affiliations:** School of Mechanical Engineering, University of Birmingham, Birmingham, UK

**Keywords:** Intervertebral disc replacement, spine simulators, ultra-high-molecular weight polyethylene, wear rates

## Abstract

The Charité^®^ lumbar intervertebral disc replacement was subjected to wear testing in an electro-mechanical spine simulator. Sinusoidally varying compression (0.6–2 kN, frequency 2 Hz), rotation (±2°, frequency 1 Hz), flexion–extension (6° to −3°, frequency 1 Hz) and lateral bending (±2°, frequency 1 Hz) were applied out of phase to specimens immersed in diluted calf serum at 37 °C. The mass of the ultra-high-molecular weight polyethylene component of the device was measured at intervals of 0.5, 1, 2, 3, 4 and 5 million cycles; its volume was also measured by micro-computed tomography. Total mass and volume losses were 60.3 ± 4.6 mg (mean ± standard deviation) and 64.6 ± 6.0 mm^3^. Corresponding wear rates were 12.0 ± 1.4 mg per million cycles and 12.8 ± 1.2 mm^3^ per million cycles; the rate of loss of volume corresponds to a mass loss of 11.9 ± 1.1 mg per million cycles, that is, the two sets of measurements of wear agree closely. Wear rates also agree closely with measurements made in another laboratory using the same protocol but using a conventional mechanical spine simulator.

## Introduction

This technical note presents results on the wear of the Charité^®^ intervertebral disc replacement (DePuy Spine Inc., Raynham, MA, USA) obtained using an electro-mechanical simulator. Replacement of an intervertebral disc in the lumbar spine is one possible surgical treatment for patients with chronic low back pain.^1^ The Charité device consists of two metal plates, fixed to bone above and below the joint, with a socket in each; an ultra-high-molecular weight polyethylene (UHMWPE) spacer separates the plates and protrudes into the sockets, forming a ball-and-socket joint.^2^ There are several published studies of wear of the UHMWPE spacer in this device, obtained using conventional mechanical simulators;^3,4^ later studies have focussed on regional wear of the spacer.^4,5^ The Bose SDWS-1 Spine Simulator (Bose Corporation, Minnesota, MN, USA) has 6 degrees of freedom and is driven by electro-mechanical motors. It has been used to investigate friction^6–8^ and wear^9^ in possible candidates for improved disc replacement devices; it has also been used to determine wear in the NuNec^®^ device intended to replace intervertebral discs in the cervical spine^10^ and the flexural properties of an elastomeric device that functions as a flexible coupling rather than as a ball-and-socket device.^11^

The main purpose of this technical note is to determine whether results obtained by the electro-mechanical simulator are comparable to those using a conventional mechanical simulator. It also provides further information on the wear of the Charité device. Comparing the results from the two types of simulator is important for two reasons. First, the electro-mechanical simulator has been used in fundamental studies of friction that could not be performed with a conventional wear simulator.^6–8^ Second, it has been used to measure the behaviour of devices that have not been investigated by conventional mechanical simulators or testing machines. Investigation of the wear of a device that has been investigated using a conventional simulator provides a method for validating the performance of the new electro-mechanical simulator. In a study of dental restorative materials, significantly different results were obtained using different simulators.^12^ There is no good reason for preferring one type of simulator over the other; however, mechanical simulators have been used in the earlier work on other artificial disc replacements^13–16^ and have a long history of successful application, especially in determining the wear of artificial hip replacements.^17^ However, unlike the Bose SDWS-1 Spine Simulator, most conventional simulators test several devices at once that can lead to artefacts.^18^

## Materials and methods

### Specimens

Four samples of the Charité device were investigated, which is a similar number used in some previous wear tests.^19,20^ They were the smallest size available (Size 1) with the thinnest UHMWPE spacer (7.5 mm) and parallel plates (0°). The metal marker rings were removed from the spacer for comparability with published tests.^4,5,21^ The ring detects the location of the implanted UHMWPE core. It does not affect the device’s mechanical properties. The devices were not washed before testing since they had been supplied sterile for implantation, packed in double-sealed plastic packages. However, an air spray (RS Components Ltd, Northants, UK) was used to remove any dust that could have settled after opening the packs.

### Mechanical testing

**Figure 1.**
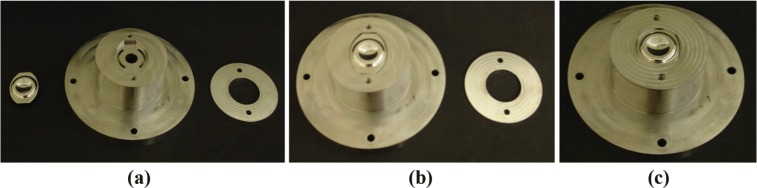
Attachment of a plate of the Charité^®^ device to the spine simulator: (a) the plate (left-hand side) of the device, the main body (centre) of the fixture that is attached to the base or actuator and the lid (right-hand side); (b) the plate fitted into the recess of the body and (c) the plate secured in the recess by the lid.

The metal plates of the device were attached to the testing machine by the fixture, shown in Figure 1, manufactured from grade 316 stainless steel. This fixture consists of two lids and two main bodies. Each plate was inserted snugly into a recess in a body (clearance ± 0.1 mm) and secured in place by a lid, as shown in Figure 1. The bodies were attached to the base and actuator of the simulator with the plates of the device horizontal. UHMWPE spacers were soaked in distilled water at 37 °C for 2 weeks before testing to stabilise fluid uptake.^22^

Tests were performed using a Bose SDWS-1 Spine Simulator (see above); a Bose ElectroForce^®^ 3330 Series II Testing Machine was used as a ‘load soak’ control station. Wear tests were performed in accordance with BS ISO 18192-1,^23^ except when stated otherwise. Sinusoidally varying compression (0.6–2 kN, frequency 2 Hz), rotation (±2° about the vertical axis, frequency 1 Hz), flexion–extension (corresponding to bending the spine forwards and backwards, +6° to −3°, frequency 1 Hz) and lateral bending (corresponding to sideways bending of the spine, ±2°, frequency 1 Hz) were applied out-of-phased as described by the standard.

During testing, the devices were immersed in diluted calf serum (to a protein concentration of 15 ± 2 g/L) with distilled water; sodium azide (0.3 g/L) was added to reduce bacterial contamination at a temperature of 37 °C. Three devices were tested for 5 million cycles; the fourth was a control sample that was subjected to load soak conditions of sinusoidal compression (0.6–2 kN, frequency 2 Hz). The purpose of this control was to monitor liquid uptake or loss during the test. Specimens were cleaned and measured at intervals of 0.5, 1, 2, 3, 4 and 5 million cycles. Specimens were cleaned as per BS ISO 14242-2:2000.^24^ and the Standard Operating Protocol for Spine Wear Simulator Studies (SOP01.6) of the Institute of Medical and Biological Engineering, University of Leeds.

### Measuring wear

The mass of each spacer was measured six times with a precision of 0.2 mg using a standard laboratory balance (Ohaus GA200D; Ohaus Scales and Balances, Thetford, Norfolk, UK). The volume of each spacer was measured by micro-computed tomography using a SkyScan-1172 high-resolution system (SkyScan, Kontich, Belgium) in the School of Dentistry, University of Birmingham.

## Results

**Figure 2.**
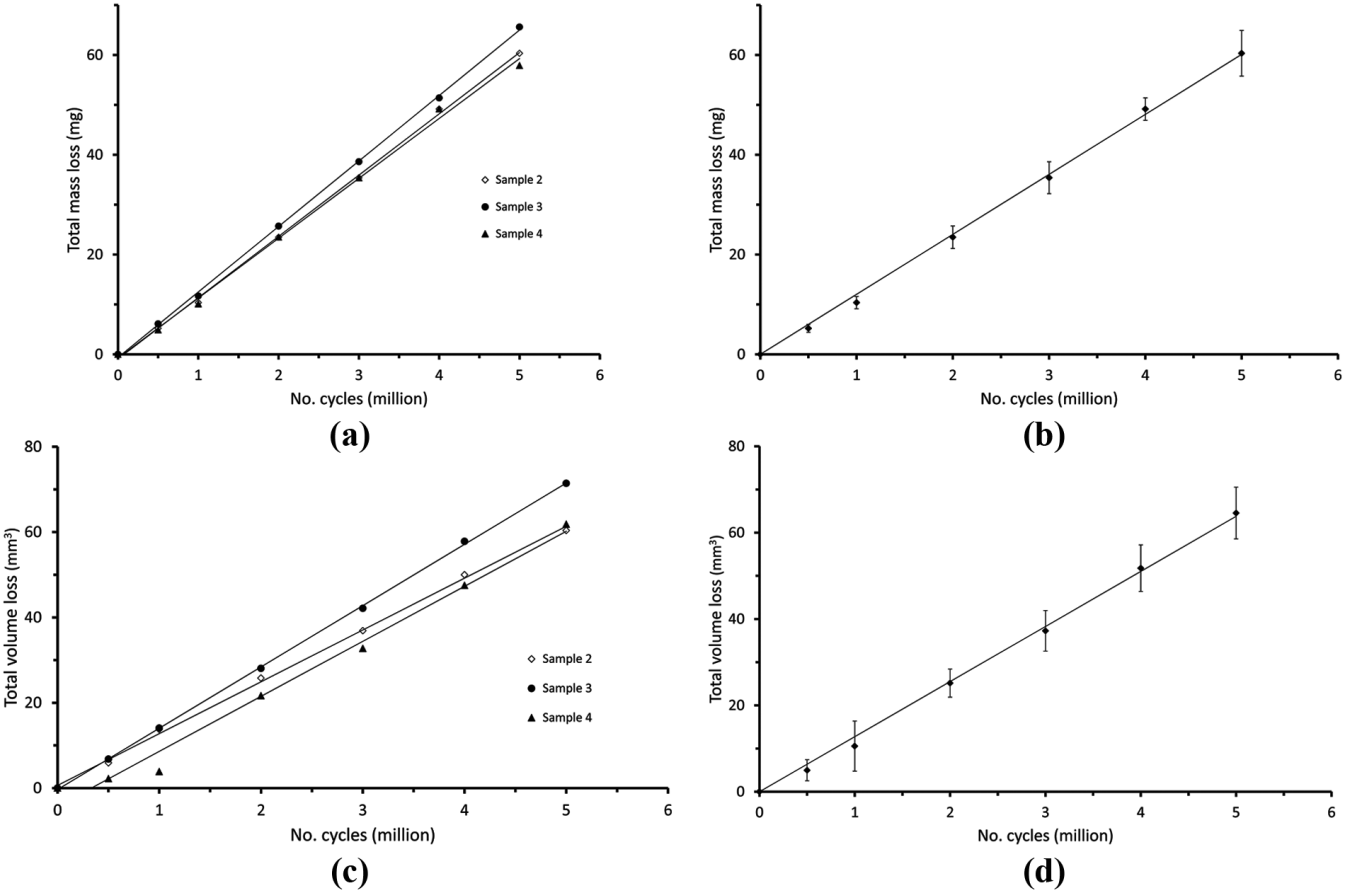
Wear of the UHMWPE spacer of the Charité^®^ device represented as (a) mass loss for each sample, (b) average mass loss, (c) volume loss for each sample and (d) average volume loss, as a function of the number of cycles. In both average cases, a regression line is fitted through the points (*R*^2^ = 0.99, *p* = 0.00) and error bars represent standard deviations. For the individual sample plots, regression lines are fitted through the points. Results for the load soak control (Sample 1) are not shown; the behaviour of this sample is described in section ‘Results’.

Figure 2(a) and (b) shows that there was a linear mass loss for the three tested samples during the 5 million test cycles. The total mass lost was 60.3 ± 4.6 mg (mean ± standard deviation) and the wear rate was 12.0 ± 1.4 mg per million cycles. The control sample appeared to show a total mass increase of 0.5 mg, that is, a mass increase of 0.1 mg every million cycles which was the longest period between measurements. Since measurements of mass increase were less than the precision of the balance (0.2 mg), they were considered to be negligible.

Figure 2(c) and (d) shows that there was a linear volume loss for the three tested samples during the 5 million test cycles. The total volume loss was 64.6 ± 6.0 mm^3^ and the wear rate was 12.8 ± 1.2 mm^3^ per million cycles. Multiplication by the density of UHMWPE (0.931 g/cm^3^)^21^ enables the volume loss and wear rate to be converted into mass loss and wear rate; the results are 60.1 ± 5.6 and 11.9 ± 1.1 mg per million cycles, respectively. The Bland–Altman statistical test^25^ shows no significant difference (*p* < 0.05) between the results of the two techniques.

## Discussion

The linear wear shown in Figure 2 is consistent with the published results on the Charité device.^4,5^ These published results were obtained with a mechanical spine simulator. Another study of the Charité device showed a period of running-in followed by a period of steady, slower wear.^3^ However, this study was performed using a hip simulator under different loading and angular displacement conditions as described in an earlier ASTM F2423-05^26^ standard. It is possible that the difference can be attributed to these different conditions, emphasising the need to compare the results obtained following the same standards when comparing the performance of different devices. An initial running-in period has been observed in intervertebral disc replacements with metal-on-metal articulation^8,14,16^ and in metal-on-metal hip replacement devices^27,28^ where the initial phase has been attributed to wear of surface asperities. This initial wear, leading to reduced friction, has been called ‘self-polishing’.^28,29^ Previous studies of wear of intervertebral disc replacement devices, in which UHMWPE articulates with metal, have reported evidence for abrasive and adhesive wear.^5,30,31^ Light scratches were just visible on both articulating surfaces during the tests reported here and could be consistent with abrasive wear. In this study, the UHMWPE surfaces developed a glossy appearance that is consistent with adhesive wear.

Reported wear rates for the Charité device were 13.1 ± 1.1^4^ and 12.9 ± 2.5 mm^3^ per million cycles.^5^ These results are very close to those obtained here, using an electro-mechanical simulator, of 12.8 ± 1.2 mm^3^ per million cycles. Furthermore, the wear rate of the UHMWPE component of the Pro-Disc^®^ device has been reported to be 11.6 ± 1.2 mg per million cycles,^16^ that is, 12.5 ± 1.3 mm^3^ per million cycles, which is very close to the UHMWPE wear rate reported here (12.0 ± 1.4 mg per million cycles).

The tests in this study were performed to 5 million cycles. Although BS ISO 18192-1^23^ suggests testing to 10 million cycles, it has been shown that the wear rate of total hip replacements reaches steady state after the first few million cycles^16,32^ and the same was found for this study. Testing to 10 million cycles would not have changed the wear rates under steady-state conditions.
